# Antiplatelet Activity of Acylphloroglucinol Derivatives Isolated from *Dryopteris crassirhizoma*

**DOI:** 10.3390/molecules24122212

**Published:** 2019-06-13

**Authors:** Nam-Hui Yim, Jung-Jin Lee, BoHyoung Lee, Wei Li, Jin Yeul Ma

**Affiliations:** 1 Korean Medicine (KM) Application Center, Korea Institute of Oriental Medicine, 70 Cheomdan-ro, Dong-gu, Daegu 41062, Korea; nhyim23@kiom.re.kr (N.-H.Y.); liwei1986@kiom.re.kr (W.L.); 2 Myungmoon Bio. Co., Ltd., 180 Yuram-ro, Dong-gu, Daegu 41059, Korea; mhjj3998@naver.com; 3 ViroMed Co., Ltd., Seoul National University, Gwanak-ro, Gwanak-gu, Seoul 151-747, Korea; bhlee@viromed.co.kr

**Keywords:** *Dryopteris crassirhizoma*, antiplatelet activity, acylphloroglucinol, butyryl-3-methylphloroglucinol

## Abstract

Platelets are an important component of the initial response to vascular endothelial injury; however, platelet dysfunction induces the acute clinical symptoms of thrombotic disorders, which trigger severe cardiovascular diseases such as myocardial infarction, ischemia, and stroke. In this study, we investigated the *Dryopteris crassirhizoma*’s antiplatelet activity. A water extract of *D.* *crassirhizoma* (WDC) was partitioned into dichloromethane (DCM), ethyl acetate, *n*-butyl alcohol, and water. Among these four fractions, the DCM fraction potently inhibited the collagen-stimulated platelet aggregation in a concentration-dependent manner. From this fraction, five different acylphloroglucinol compounds and one flavonoid were isolated by activity-guided column chromatography. They were identified by comparing their mass, ^1^H-, and ^13^C-NMR spectral data with those reported in the literature. Quantifying the six compounds in WDC and its DCM fraction by high-performance liquid chromatography (HPLC) revealed that butyryl-3-methylphloroglucinol (compound **4**) was the most abundant in these samples. Additionally, butyryl-3-methylphloroglucinol showed the strongest inhibitory activity in the collagen- and arachidonic acid (AA)-induced platelet aggregation, with inhibition ratios of 92.36% and 89.51% in the collagen and AA-induced platelet aggregation, respectively, without cytotoxicity. On the active concentrations, butyryl-3-methylphloroglucinol significantly suppressed the convulxin-induced platelet activation. Regarding the structure–activity relationships for the five acylphloroglucinol compounds, our results demonstrated that the functional butanonyl, methoxy, and hydroxy groups in butyryl-3-methylphloroglucinol play important roles in antiplatelet activity. The findings indicate that acylphloroglucinols, including butyryl-3-methylphloroglucinol from *D.* *crassirhizom*, possess an antiplatelet activity, supporting the use of this species for antiplatelet remedies.

## 1. Introduction

The rhizome of *D. crassirhizoma*, called “Gwanjung” in Korea (“Guan Zhong” in China and “Oshida” in Japan), has been extensively used to treat parasitic infestation, hemorrhage, epidemic flu, cold, and cancer [1,2,3,4]. Previous studies showed that the *D. crassirhizoma* rhizome exhibits various pharmacological activities, such as antioxidant [5], antiobesity [6], antiparasite, antiviral [7], antibacterial [8], anti-inflammatory [9], and anticancer effects [10]. In particular, our previous study of the water extract from the *D. crassirhizoma* rhizome identified its anti-osteoporotic effects from studies on in vitro and in vivo systems [11]. Phytochemical studies of *D. crassirhizoma* have also revealed various compounds, including flavonoids, triterpenes, and phloroglucinols [8,12,13,14]. Acylphloroglucinol, an acyl moiety of phloroglucinol, which is enriched in *Dryopteris* species, has various pharmacological properties, including antibacterial, anticancer, antioxidant, and anti-inflammatory effects [6,15]. However, the antiplatelet activity of acylphloroglucinol derivates isolated from *D. crassirhizoma* has not been reported.

Platelets are an important component of the initial response to vascular endothelial injury. They usually accumulate at the injury site, which is essential for the formation of hemostatic plugs [16,17]. However, this response contributes to pathogenic thrombus formation, which is associated with thrombotic disorders’ acute clinical symptoms. Such pathogenesis is evident in patients with cardiovascular diseases, including myocardial infarction, ischemia, and stroke [17,18,19]. Platelets gather at the site of injury and induce the adhesion of glycoprotein (GP) Ib and GPVI to von Willebrand factor and collagen, respectively [19]. Among these platelet activators, collagen, acting as a physiological agonist, induces rapid changes in the membrane phospholipid composition by activating phospholipases (PLs) C and A_2_ [20,21,22]. Membrane changes include arachidonic acid (AA) release, catalyzed by PLA_2_, and Ca^2+^ mobilization, in turn triggering protein kinase (PK) C activation by 1,2-dicacylglycerol (DAG). AA can be metabolized by cyclooxygenase (COX) and thromboxane (TX) synthase to yield prostaglandins and TXA_2_, both of which are potent platelet agonists [21,23]. Thus, the inhibition of platelet activation by collagen may prevent thrombotic disorder development in patients with cardiovascular disease.

In this study, the constituents in the dichloromethane (CH_2_Cl_2,_ DCM) fraction of WDC were isolated and investigated for their inhibitory activities against collagen-induced platelet aggregation in vitro.

## 2. Results

### 2.1. The Effects of WDC and its Fractions on Collagen-Stimulated Platelet Aggregation

**Figure 1:**
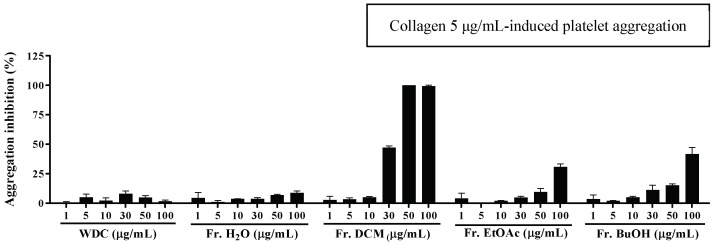
Effects of the water extract of *D. crassirhizoma* (WDC) and four fractions from WDC on the collagen-stimulated platelet aggregation. A washed rabbit platelet suspension was treated with various concentrations of the fractions for 3 min, and the effect on the collagen (5 μg/mL)-induced platelet aggregation was measured. The extent of the platelet aggregation is expressed as a percentage of the control ± standard error.

To verify the antiplatelet activity of WDC extracts, namely water crude, H_2_O, dichloromethane (CH_2_Cl_2_), ethyl acetate (EtAOc), and butanol (BuOH) fractions, we investigated WDC extracts’ effect on the collagen-stimulated platelet aggregation. Among these fractions, at the same concentration, the CH_2_Cl_2_ fraction potently inhibited the collagen-stimulated platelet aggregation in a concentration-dependent manner (IC_50_ = 30.48 μg/mL) (Figure 1). Hence, based on the above results, we investigated the DCM fraction’s active constituents.

### 2.2. Isolation of Active Compounds from DCM Fraction of WDC

**Figure 2:**
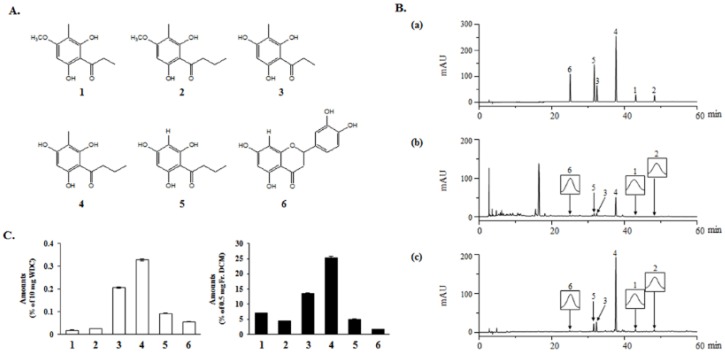
Identification of compounds isolated from the DCM fraction of WDC. (**A**) Chemical structures of the six isolated compounds. (**B**) Similarity analysis of the isolated compounds in the standard mixture (a), WDC (b), and its DCM fraction (c) at 280 nm by a HPLC-DAD analysis. (**C**) Quantification of the six isolated compounds in WDC and its DCM fraction: (**1**) aspidinol P, (**2**) aspidinol B, (**3**) propionyl-3-methylphloroglucinol, (**4**) butyryl-3-methylphloroglucinol, (**5**) butyrylphloroglucinol, and (**6**) eriodictyol.

The DCM fraction was further separated by column chromatography, using silica gel and preparative HPLC, and six compounds were isolated. Their chemical structures were determined by an extensive analysis of the physicochemical and spectroscopic data, such as ^1^H-NMR, ^13^C-NMR, and LC/MS analyses, as well as by comparisons with the literature. This led to their identification as five acylphloroglucinols, namely aspidinol P (**1**) [24], aspidinol B (**2**) [25], propionyl-3-methylphloroglucinol (**3**) [25], butyryl-3-methylphloroglucinol (**4**) [25], and butyrylphloroglucinol (**5**) [25], as well as one flavonoid, eriodictyol (**6**) [12,26] (Figure 2A).

### 2.3. Qualitative and Quantitative Analyses of the Isolated Compounds in WDC and Its DCM Fraction 

The six compounds isolated from the DCM fraction were simultaneously analyzed quantitatively and qualitatively using HPLC-DAD. To improve the chromatographic separation capacity, 0.1% TFA (*v*/*v*) in water (A) and acetonitrile (B) were used as mobile phases in a gradient elution system. The present chromatographic conditions were used to establish the specific HPLC retention times (*t_R_*) and the UV detection wavelengths for the six standard compounds, which were used to identify the six compounds in the DCM fraction and in WDC. As shown in Figure 2B, the six-compound mixture retention times were 25.02 (**6**), 31.62 (**5**), 32.38 (**3**), 37.68 (**4**), 43.07 (**1**), and 48.21 min (**2**) at 280 nm. Under the same conditions, the observed compounds’ retention times were 24.99 (**6**), 31.60 (**5**), 32.37 (**3**), 37.68 (**4**), 43.06 (**1**) and 48.06 min (**2**) in the DCM fraction, and 24.98 (**6**), 31.57 (**5**), 32.34 (**3**), 37.63 (**4**), 42.99 (**1**) and 48.14 min (**2**) in WDC.

The calibration data of each standard compound showed a good linearity (*R*^2^ > 0.9983–0.9999). The LOD was between 0.205 and 0.564 μg/mL, and the LOQ was between 0.683 and 1.879 μg/mL (Table S1).

The developed HPLC-based analytical method was applied to the simultaneous quantitation of the six isolated compounds in WDC and its DCM fraction (Figure 2C). The five acylphloroglucinols showed high proportions among these isolated compounds in the DCM fraction. In particular, butyryl-3-methylphloroglucinol (**4**) was the most abundant in both WDC and its DCM fraction. Eriodictyol (**6**), the flavonoid isolated from the DCM fraction, was detected at higher levels than aspidinol P (**1**) and aspidinol B (**2**) in WDC; however, it was detected at relatively low levels compared to the five acylphloroglucinols in the DCM fraction.

### 2.4. Antiplatelet Activity of Compounds Isolated from DCM Fraction of WDC

**Figure 3:**
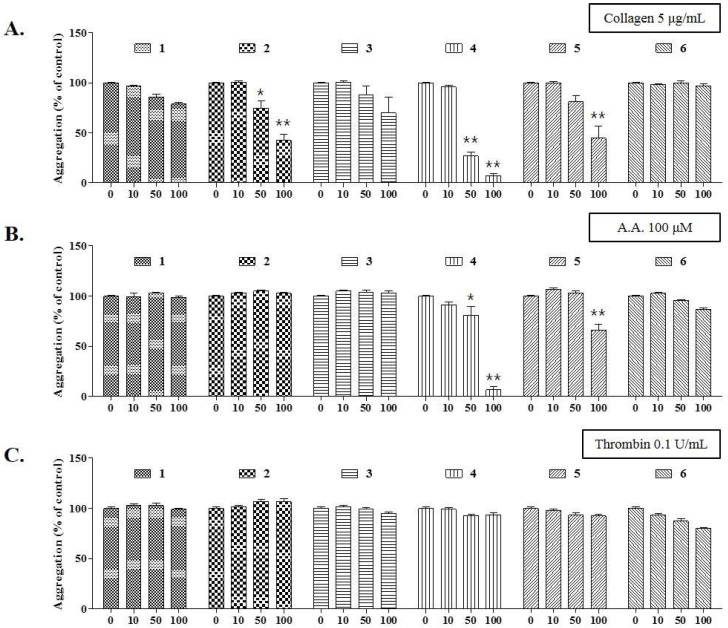
Effects of six compounds isolated from the DCM fraction on the collagen- and arachidonic acid-stimulated platelet aggregation. A washed rabbit platelet suspension was treated with various concentrations of the fractions for 3 min, and the effects of fractions A1–A6 on the (**A**) collagen (5 μg/mL)-, (**B**) arachidonic acid (AA, 100 μM)-, and (**C**) thrombin (0.1 U/mL)-induced platelet aggregation were measured. The extent of the platelet aggregation is expressed as a percentage of the control ± standard error. Significant differences from the control (untreated) are indicated by * *p* < 0.05 and ** *p* < 0.01.

**Figure 4:**
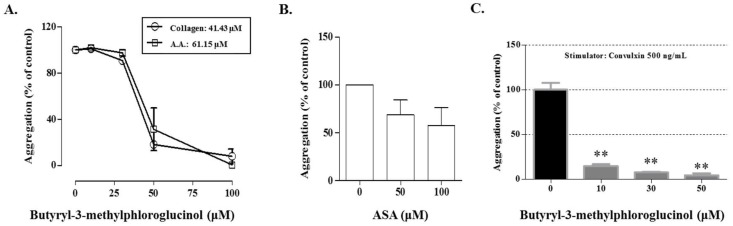
Antiplatelet effect of butyryl-3-methylphloroglucinol. (**A**) Determination of the IC_50_ values of butyryl-3-methylphloroglucinol on the collagen (5 μg/mL)- and arachidonic acid (AA, 100 μM)-induced platelet aggregation. (**B**) The effects of 50 and 100 μM ASA on the collagen (5 μg/mL)-induced platelet aggregation. (**C**) Effect of butyryl-3-methylphloroglucinol on the convulxin (500 ng/mL)-induced platelet aggregation. A washed rabbit platelet suspension was treated with various concentrations of the fractions for 3 min, and the effects of butyryl-3-methylphloroglucinol or ASA on the collagen (5 μg/mL)- and arachidonic acid (AA, 100 μM) and convulxin (500 ng/mL)-induced platelet aggregation were measured. The extent of the platelet aggregation is expressed as a percentage of the control ± standard error. Significant differences from the control (untreated) are indicated by ** *p* < 0.01.

To confirm the antiplatelet activity, the effects of these compounds were investigated on the collagen-, AA-, or thrombin-stimulated platelet aggregation. As shown in Figure 3, aspidinol B (**2**), butyryl-3-methylphloroglucinol (**4**), and butyrylphloroglucinol (**5**) markedly suppressed the collagen-induced platelet aggregation. In particular, at a concentration of 100 μM, butyryl-3-methylphloroglucinol (**4**) inhibited the collagen and AA-induced platelet aggregation by 92.36% and 89.51%, respectively, without cytotoxicity (Figure 3A,B). At the same concentrations, aspidinol P (**1**) and propionyl-3-methylphloroglucinol (**3**) weakly inhibited the collagen-induced platelet aggregation only. The tested compounds had no antiplatelet effects (Figure 3C) on inducing the platelet aggregation with thrombin. Eriodictyol (**6**) did not show antiplatelet activity under any induction conditions. Under the same conditions, butyryl-3-methylphloroglucinol (**4**) showed IC_50_ values of 41.43 and 61.15 μM in collagen (5 μg/mL)- and AA (100 μM)-induced the platelet aggregations in vitro, respectively, whereas the IC_50_ values of acetylsalicylic acid (ASA) were determined to be 100 μM or more for the collagen-induced platelet aggregation, which represented a stronger antiplatelet activity than that of ASA (Figure 4). In addition, to demonstrate the mechanism of butyryl-3-methylphloroglucinol, we investigated the effect on convulxin, which is a specific stimulator against the collagen receptor GPVI-induced platelet aggregation [27]. Butyryl-3-methylphloroglucinol potently inhibited the platelet activation at a concentration of 10 μM or more.

## 3. Discussion

Several studies have reported the isolation of bioactive constituents such as phloroglucinols, kaempferol-rhamnosides and -acetylrhamnosides, and dimethylflavanones in *D. crassirhizoma* [12,14]. Among them, phloroglucinol derivatives are present in various *Dryopteris* species, and these derivatives confer typical bioactivities. Previous studies demonstrated the antiplatelet activity of phloroglucinol by inhibiting blood coagulation and inflaming the persistent vascular wall. Phloroglucinol showed antithrombotic and profibrinolytic activities by reducing the generation of thrombin and the coagulation factor Xa (FXa) in endothelial cells [28]. In platelets, phloroglucinol attenuated the AA-induced platelet aggregation, which is related to suppressing inflammation-mediated factors, such as cyclooxygenase (COX) and reactive oxygen species, through regulating the mitogen-activated protein kinase pathway [9,29]. According to a study analyzing the total phloroglucinols from several solvent fractions of *D. crassirhizoma* 70% aqueous ethanol extract, the chloroform fraction had the highest total phloroglucinol concentrations and showed the highest nematicidal activity against root-knot nematodes [30,31]. We also demonstrated that the dichloromethane fraction (Fr. DCM) from *D. crassirhizoma* water extract displayed the strongest inhibitory activity among four different solvent fractions in collagen-induced platelet aggregation conditions. These results demonstrate that the antiplatelet activity of Fr. DCM appears to be associated with high phloroglucinol concentrations, which may lead to an antiplatelet drug development.

Phloroglucinols are classified into monomeric, dimeric, trimeric, tetrameric, and phlorotannins, and acylphloroglucinols are a subclass of monomeric phloroglucinols [32]. In this study, we explored five acylphloroglucinols to demonstrate *D. crassirhizoma*’s antiplatelet activity. In terms of the platelet aggregation, butyryl-3-methylphloroglucinol (**4**) and butyrylphloroglucinol (**5**) dose-dependently inhibited the platelet activation stimulated by collagen or AA, but not by thrombin. Moreover, butyryl-3-methylphloroglucinol significantly suppressed the convulxin-induced platelet activation; this result shows the specific inhibition against the GPVI receptor, which is a major receptor of collagen [33,34]. As a possible mechanism behind this inhibition, butyryl-3-methylphloroglucinol (**4**) and butyrylphloroglucinol (**5**) may exert an antiplatelet activity by suppressing the collagen-induced phospholipase (PL) Cγ-mediated signal transduction, including the intracellular calcium mobilization and thromboxane A_2_ and prostaglandin conversion from AA [35,36], rather than the PLCβ-mediated signal transduction induced by thrombin [37]. In contrast, aspidinol B (**2**) showed an inhibitory effect only against the collagen-induced platelet aggregation, which indicates an antiplatelet effect through the PLCγ-mediated signal transduction. These results indicate that aspidinol B (**2**), butyryl-3-methylphloroglucinol (**4**), and butyrylphloroglucinol (**5**) may selectively affect the collagen- and arachidonic acid-mediated platelet aggregation.

**Figure 5:**
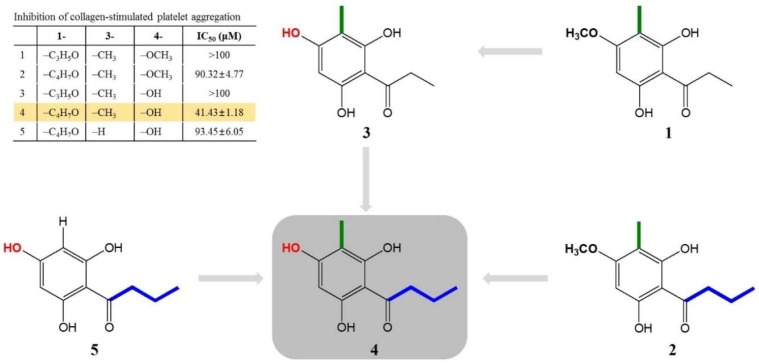
Identification of the structure–activity relationship for the antiplatelet activity in compounds isolated from the DCM fraction of WDC.

Interestingly, the structural properties and antiplatelet activities of five compounds (**1**–**5**) allowed us to make inferences regarding the structure–activity relationship (SAR). When we compared aspidinol P (**1**) and butyryl-3-methylphloroglucinol (**4**), we inferred that butyryl-3-methylphloroglucinol (**4**) had a strong activity because of a butanonyl group located at C-1, while aspidinol P (**1**) contains a propanonyl group, resulting in no activity (IC_50_ > 100 μM). Therefore, the presence of a butanonyl group at C-1 is likely to be a key functional element. On the other hand, compared with aspidinol B (**2**), butyryl-3-methylphloroglucinol (**4**) contains a hydroxy group at C-4, while aspidinol B (**2**) contains a methoxy group, which also resulted in a weak activity (IC_50_ = 90.32 μM). This suggests that the hydroxy group plays an important role in the antiplatelet activity. Furthermore, comparing the IC_50_ values and structures of butyryl-3-methylphloroglucinol (**4**) and butyrylphloroglucinol (**5**), the methyl group at C-3 was also considered a key functional element. In conclusion, a butanonyl group (C-1), a methoxy group (C-4), and a hydroxy group (C-3) were identified as functional groups in the present SAR study, and the observed activity was proportional to the number of functional groups of each isolated compound (Figure 5). In the present study, butyryl-3-methylphloroglucinol (**4**) potently showed an antiplatelet effect, so we are conducting further studies to demonstrate that the antiplatelet efficacy of butyryl-3-methylphloroglucinol (**4**) occurs through an antagonistic activity against a major collagen receptor.

## 4. Materials and Methods

### 4.1. Chemicals and Instruments

Collagen, arachidonic acid (AA), and thrombin were purchased from Chrono-Log Co. (Havertown, PA, USA). Trifluoroacetic acid (TFA) and acetylsalicylic acid (ASA, aspirin) were purchased from Sigma-Aldrich Co. (St. Louis, MO, USA). Acetonitrile of high-performance liquid chromatography (HPLC) grade was obtained from J.T. Baker Inc. (Philipsburg, NJ, USA), and deionized water was prepared using an ultrapure water production apparatus (Millipore, Billerica, MA, USA).

A high-performance liquid chromatography (HPLC) for the preparation of the compound was performed using a Waters HPLC system (600 pump, 600 controller, and 996 photodiode array detector). The ^1^H-NMR and ^13^C-NMR spectra were obtained using a JNM AL 400 MHz NMR spectrophotometer (Jeol Ltd., Tokyo, Japan). The LC/MS spectra for identifying the molecular weights of single compounds were performed on an Agilent 1100+G1958B system (Agilent Technologies, Inc., Santa Clara, CA, USA). Column chromatography was performed on silica gel (Kieselgel 60, 70–230 mesh, Merck, Kenilworth, NJ, USA) and YMC RP-18 resins (30–50 μm, Fujisilisa Chemical Ltd., Aichi, Japan). A thin layer chromatography (TLC) was performed on DC-Alufolien 60 silica-gel F_254_ (Merck 1.05554.0001) or DC Platten RP_18_ F_254s_ (Merck 1.15685.0001) plates. Compounds were detected under UV light and then visualized by spraying the plates with 10% sulfuric acid reagent followed by heating for 1 min.

### 4.2. Isolation of Six Compounds from DCM Fraction of WDC

The rhizomes of *Dryopteris crassirhizoma* were purchased from Gangwon province in Korea. The reference specimen was deposited at the Korea Institute of Oriental Medicine (KIOM). The dried powder of *D. crassirhizoma* (1 kg) was refluxed with water for 3 h (3 × 6 L), and the extract was freeze-dried (WDC). WDC (100 g), suspended in water (1000 mL), was continuously extracted with DCM_,_ ethylacetate (EtOAc) and water-saturated butanol (BuOH). The DCM soluble fraction (1.1 g) was chromatographed on a silica gel column (CHCl_3_: MeOH 100:1→0:1) to yield six fractions (Fr. A1–A4). In Fr. A1, compounds **1** (10.1 mg) and **2** (5.4 mg) were obtained by preparative HPLC (MeOH:H_2_O-55:45). Fraction A2 was separated by silica gel CC using CH_2_Cl_2_: MeOH (40: 1, *v*/*v*) to obtain the four fractions (Fr. B1–Fr. B3). Compounds **3** (3.7 mg) and **4** (44.3 mg) were obtained from Fr. B2 by preparative HPLC (ACN: H_2_O-70:30). Fraction A3 was separated by RP-18 CC using MeOH:H_2_O (1:1. *v*/*v*) and obtained by preparative HPLC (ACN:H_2_O-25:75) to obtain compound **5** (7 mg). Compound **6** (17.9 mg) was obtained by preparative HPLC (MeOH:H_2_O-50:50). The purity of all isolates (1–6) was > 98% by using HPLC. The structures were identified by comparing the spectral data (UV, MS, ^1^H- and ^13^C-NMR) with previous literature.

1-(2,6-dihydroxy-4-methoxy-3-methyl-phenyl)-propan-1-one (**1**, Aspidinol P)—Red powder; LC-ESI MS (positive mode) *m*/*z*: 211.1 [M + H]^+^; 1H-NMR (400 MHz, MeOD) δ: 5.98 (1H, s, H-5), 3.79 (3H, s, -OCH3), 3.09 (2H, t, *J* = 7.2 Hz, H-9), 1.89 (3H, s, H-7), 1.13 (3H, t, *J* = 7.2 Hz, H-10); ^13^C-NMR (100 MHz, MeOD) δ: 208.3 (C=O), 164.9 (C-6), 163.6 (C-4), 161.9 (C-2), 104.6 (C-1), 90.9 (C-5), 55.8 (-OCH3), 38.2 (C-9), 19.1 (C-10), 7.2 (C-7).

1-(2,6-dihydorxy-4-methoxy-3-methyl-phenyl)-butan-1-one (**2**, Aspidinol B)—Red powder; LC-ESI MS (positive mode) *m*/*z*: 225.1 [M + H]^+^; 1H-NMR (400 MHz, MeOD) δ: 5.99 (1H, s, H-5), 3.80 (3H, s, -OCH3), 3.04 (2H, t, *J* = 7.2 Hz, H-9), 1.90 (3H, s, H-7), 1.68 (2H, q, *J* = 7.2 Hz, H-10), 0.97 (3H, s, C-11); ^13^C-NMR (100 MHz, MeOD) δ: 207.8 (C=O), 164.9 (C-6), 163.7 (C-4), 161.9 (C-2), 104.6 (C-1), 90.9 (C-5), 55.9 (-OCH3), 47.0 (C-9), 19.4 (C-10), 7.2 (C-7).

1-(2,4,6-trihydroxy-3-methyl-phenyl)-propan-1-one (**3**, propionyl-3-methylphloroglucinol)—Red powder; LC-ESI MS (negative mode) *m*/*z*: 195.1 [M − H]^−^; ^1^H-NMR (400 MHz, MeOD) δ: 5.88 (1H, s, H-5), 3.07 (2H, t, *J* = 7.5 Hz, H-9), 1.90 (3H, S, H-7), 1.13 (3H, t, *J* = 7.5 Hz, H-10); ^13^C-NMR (100 MHz, MeOD) δ: 208.0 (C=O), 164.9 (C-6), 163.7 (C-4), 161.4 (C-2), 103.6 (C-1), 94.8 (C-5), 38.1 (C-9), 9.4 (C-10), 7.4 (C-7).

1-(2,4,6-trihydroxy-3-methylphenyl)butan-1-one (**4**, butyryl-3-methylphloroglucinol)—Red powder; LC-ESI MS (positive mode) *m*/*z*: 209.2 [M − H]^−^; ^1^H-NMR (400 MHz, MeOD) δ: 5.87 (1H, s, H-5), 3.01 (2H, t, *J* = 7.6 Hz, H-9), 1.90 (3H, s, H-7), 1.68 (2H, m, H-10), 0.96 (3H, s, H-11); ^13^C-NMR (100 MHz, MeOD) δ: 207.4 (C=O), 164.9 (C-6), 163.7 (C-4), 161.4 (C-2), 103.6 (C-1), 94.8 (C-5), 46.9 (C-9), 19.6 (C-10), 14.4 (C-11), 7.4 (C-7).

1-(2,4,6–trihydroxyphenyl)butan-1-one (**5**, butyrylphloroglucinol)—Red powder; LC-ESI MS (negative mode) *m*/*z*: 195.1 [M − H]^−^; 1H-NMR (400 MHz, MeOD) δ: 5.79 (1H, s, H-5), 5.79 (1H, s, H-5), 3.01 (2H, t, *J* = 7.4 Hz), 1.68 (2H, m, H-9), 0.96 (3H, s, H-10); ^13^C-NMR (100 MHz, MeOD) δ: 207.4 (C=O), 166.0 (C-4), 165.9 (C-2), 165.9 (C-6), 105.5 (C-1), 95.8 (C-3), 95.8 (C-5), 46.9 (C-8), 19.6 (C-9), 14.5 (C-10).

3′,4′,5,7-Tetrahydroxyflavanone (**6**, Eriodictyol)—Yellow powder; LC-ESI MS (negative mode) *m*/*z*: 287.1 [M − H]^−^; 1H-NMR(400 MHz, MeOD) δ: 1H-NMR (400 MHz, MeOD) δ: 5.89 (1H, d, *J* = 2.0, H-6), 5.84 (1H, d, *J* = 2.0 Hz, H-8), 5.28 (1H, dd, *J* = 3.1, 12.8 Hz, H-2), 3.09 (1H, d, *J* = 12.8, H-3), 2.71 (1H, dd, *J* = 3.1, 17.2 Hz, H-3), 6.91 (1H, s, H-2′), 6.78 (1H, s, H-5′), 6.78 (1H, s, H-6′); ^13^C-NMR (100 MHz, MeOD) δ: 197.8, 168.4, 165.5, 164.9, 146.9, 146.5.

### 4.3. Chromatographic Conditions

The analyses of the compounds isolated from the DCM fraction of WDC were carried out via an HPLC analysis using a Dionex UltiMate 3000 system equipped with a binary pump, auto-sampler, column oven and diode array UV/VIS detector (DAD, Thermo Fisher Scientific, CA, USA). The chromatograms of the detector were registered using a Chromeleone software system (Ver. 7, ThermoFisher Scientific, Waltham, MA, USA). The chromatographic separation was achieved on an Acclaim^®^ C_18_ column (4.6 × 250 mm, 5 μm, Thermo Scientific) using 0.1% TFA water (*v*/*v*); solvent A and acetonitrile; solvent B as the mobile phases at a flow rate of 1 mL/min. The HPLC elution condition was optimized as follows: 0–60 min, 10–70% B; 60–70 min, 100% B. The column oven and auto-sampler injection volume were wet to 40 °C and 10 μL, respectively. The detected wavelength was set at 280 nm and the total run time was 70 min.

### 4.4. Validation of HPLC Method

The method validation for the selectivity, linearity, limit of detection (LOD), and limit of quantification (LOQ) in the present study was performed according to the Validation of Analytical Procedures: Text and Methodology Q2 (R1) Guideline of the International Conference on Harmonization (ICH). The standard calibration curve for the linearity assay was prepared with five different concentrations of diluted standard solutions and performed in triplicate. To demonstrate the linearity of the analytical method, calibration curves were generated by plotting the chromatographic peak area as a function of the analyte concentration. The linear regressions of these data were expressed as *Y* = *Ax* + *B*, where *A* is the slope of the calibration curve, *B* is the y-intercept of the calibration curve, *x* is the concentration of the marker components, and *Y* is the peak area. The correlation coefficients (*R*^2^) were calculated to assess the linearity. The limit of detection (LOD) and limit of quantification (LOQ) were determined at signal-to-noise (S/N) ratios of 3 and 10, respectively.

### 4.5. Animals

New Zealand white rabbits (2.5–3 kg) were purchased from Sam-Tako Animal Co. (Osan, Korea) and acclimated for 1 week at a temperature of 24 ± 1 °C and humidity of 55 ± 5%. The animals had free access to a commercial pellet diet obtained from Samyang Co. (Wonju, Korea) and drinking water. The animal studies have been carried out in accordance with the Korea Institute of Oriental Medicine Care Committee Guidelines, and were approved by the Korea Institute of Oriental Medicine Care and Use Committee. The animals were cared for in accordance with the dictates of the National Animal Welfare Law of Korea (No.14651).

### 4.6. Preparation of Washed Rabbit Platelet and Measurement of Platelet Aggregation In Vitro 

Blood was withdrawn from the ear artery of the male New Zealand white rabbits and collected into 0.15 (*v*/*v*) of anticoagulant citrate dextrose (ACD) solution that contained 0.8% citric acid, 2.2% trisodium citrate, and 2% dextrose (*w*/*v*). Washed platelets were prepared as previously described [38]. The platelet aggregation was measured with an aggregometer (Chrono-Log Co., Havertown, PA, USA) according to Born’s turbidimetry method [39]. Briefly, the washed platelet suspension was incubated at 37 °C in the aggregometer with stirring at 1200 rpm, and then various concentrations of WDC and its solvent fractions, including DCM-, EtOAc-, BuOH-, and H_2_O-soluble fractions, and isolated compounds from the DCM fraction, respectively. After 3 min of pre-incubation, the platelet aggregation was induced by the addition of collagen (5 µg/mL), arachidonic acid (100 µM) and thrombin (0.1 U/mL), respectively. Then extent of the platelet aggregation is expressed as a percentage of the control.

### 4.7. Statistical Analysis

The results are expressed as means ± SEM and were analyzed using an analysis of variance (ANOVA). A value of *p* < 0.05 was taken to indicate a statistical significance.

## 5. Conclusions

In this study, we revealed that the DCM fraction from WDC contains acylphloroglucinol compounds, which enhance antiplatelet activities by inhibiting the platelet aggregation. We also found that butyryl-3-methylphloroglucinol from the DCM fraction strongly inhibited the collagen- and AA-induced platelet aggregation compared to other acylphloroglucinol compounds. Based on the SAR study, we interpreted that this is related to the antiplatelet efficacy. Therefore, we suggest that acylphloroglucinols, including butyryl-3-methylphloroglucinol from *D. crassirhizoma*, possess an antiplatelet activity, which could contribute to the use of *D. crassirhizoma* for antiplatelet remedies.

## Supplementary Materials

The following are available online, Table S1: Calibration curves of six compounds isolated from DCM fraction of WDC.
