# Supplementary material for: Antiplatelet Activity of Acylphloroglucinol Derivatives Isolated from Dryopteris crassirhizoma
Source: Molecules. 2019 Jun 13;24(12):2212. doi: 10.3390/molecules24122212 (PMC6631571; doi:10.3390/molecules24122212)
Supplement: Supplementary file 1 [file molecules-24-02212-s001.pdf]

Table S1. Calibration curves of six compounds isolated from DCM fraction of WDC.

Compound	LinearRegression Equation[a)]	Correlation coefficient (r^2)[b)]	LOD (μg/mL)	LOQ (μg/mL)
1	y = 0.0335x + 0.0388	0.9999	0.564	1.879
2	y = 0.032x + 0.0016	0.9999	0.356	1.188
3	y = 0.0652x - 0.0413	0.9996	0.442	1.473
4	y = 0.2686x - 0.2519	0.9994	0.319	1.063
5	y = 0.1507x - 0.1819	0.9991	0.307	1.024
6	y = 0.1034x - 0.2341	0.9983	0.205	0.683

a) *Y*: peak area, *x*: concentration (μg/mL); b) Regression coefficient (n=6)
